# Expression and molecular profiles of the AlkB family in ovarian serous carcinoma

**DOI:** 10.18632/aging.202716

**Published:** 2021-03-19

**Authors:** Yuan Cai, Geting Wu, Bi Peng, Juanni Li, Shuangshuang Zeng, Yuanliang Yan, Zhijie Xu

**Affiliations:** 1 Department of Pathology, Xiangya Hospital, Central South University, Changsha 410008, Hunan, China; 2 Department of Pharmacy, Xiangya Hospital, Central South University, Changsha 410008, Hunan, China; 3 National Clinical Research Center for Geriatric Disorders, Xiangya Hospital, Central South University, Changsha 410008, Hunan, China

**Keywords:** ovarian serous carcinoma, AlkB family, expression profiles, infiltrating immune cells, methylation

## Abstract

AlkB family of Fe (II) and α-ketoglutarate-dependent dioxygenases plays essential roles in development of ovarian serous carcinoma (OV). However, the molecular profiles of AlkB family in OV have not been clarified. The results indicated that the expression of ALKBH1/3/5/8 and FTO was lower in OV patients while ALKBH2/4/6/7 expression was higher. There was a strong correlation between ALKBH5/7 and pathological stage of OV patients. Kaplan-Meier plotter revealed that OV patients with high ALKBH4 level showed longer overall survival (OS). However, patients with high levels of ALKBH5/6 and FTO showed shorter OS and progression-free survival (PFS). Genetic alterations using cBioPortal revealed that the alteration rates of FTO were the highest. We also found that the functions of AlkB family were linked to several cancer-associated signaling pathways, including chemokine receptor signaling. TIMER database indicated that the AlkB family had a strong relationship with the infiltration of six types of immune cells (macrophages, neutrophils, CD8+ T-cells, B-cells, CD4+ T-cells and dendritic cells). Next, DiseaseMeth databases revealed that the global methylation levels of ALKBH1/2/3/4/5/6/7/8 and FTO were all lower in OV patients. Thus, our findings will enhance the understanding of AlkB family in OV pathology, and provide novel insights into AlkB-targeted therapy for OV patients.

## INTRODUCTION

Ovarian cancer has raised worldwide concern because it has the highest fatality rate among cancers affecting only women [1–3]. WHO statistics indicate that 225,500 women are diagnosed with ovarian cancer, and 140,200 of them die from this malignant disease annually [4]. Women in developing countries and developed countries both suffer from a high death rate from ovarian cancer. Even in developed countries, a large number of patients cannot be diagnosed at an early stage [5]. Cumulative evidence has reported that ovarian serous carcinoma (OV) has the highest mortality among all histological subtypes of ovarian cancer. As shown in a recent study, we found that the survival data have not changed since 1980 in many areas [6–8]. Consequently, molecular markers will play an essential role in diagnosing OV at an early stage and assist in the treatment of patients.

Accumulating statistics indicate that among a series of RNA modification adenosines, N6-methyladenosine (m6A) plays the most significant role in modulating numerous cellular processes of eukaryotes [9–11]. Currently, an increasing number of scientists are committed to exploring the relationship between m6A and cancers, comparing differences between various types of tumors [12–14]. At the same time, a recent study revealed that m6A is linked to the pathogenesis and development of ovarian cancer by regulating several targeted genes [15]. The main regulators of m6A RNA methylation include “writers”, “erasers” and “readers”, which are named based on their biological functions. The AlkB family of Fe (II) and α-ketoglutarate-dependent dioxygenases are “erasers” and play essential roles in RNA m6A modification [16, 17]. Furthermore, another recent study conducted by Jiang et al. indicated that the expression of ALKBH5 in ovarian cancer tissue is higher than that in normal tissue. Consequently, ALKBH expression is linked to modulating ovarian cancer [18]. In addition, ALKBH5 plays an essential role in the regulation of cancer cell proliferation and invasion [19]. However, the specific mechanisms of AlkB family members in OV development and progression still require further confirmation and research.

The aim of our study was to evaluate the biological significance of AlkB family members in OV patients using comprehensive bioinformatics databases (Supplementary Table 1).

## RESULTS

### Aberrant expression of the AlkB family in OV patients

**Figure 1:**
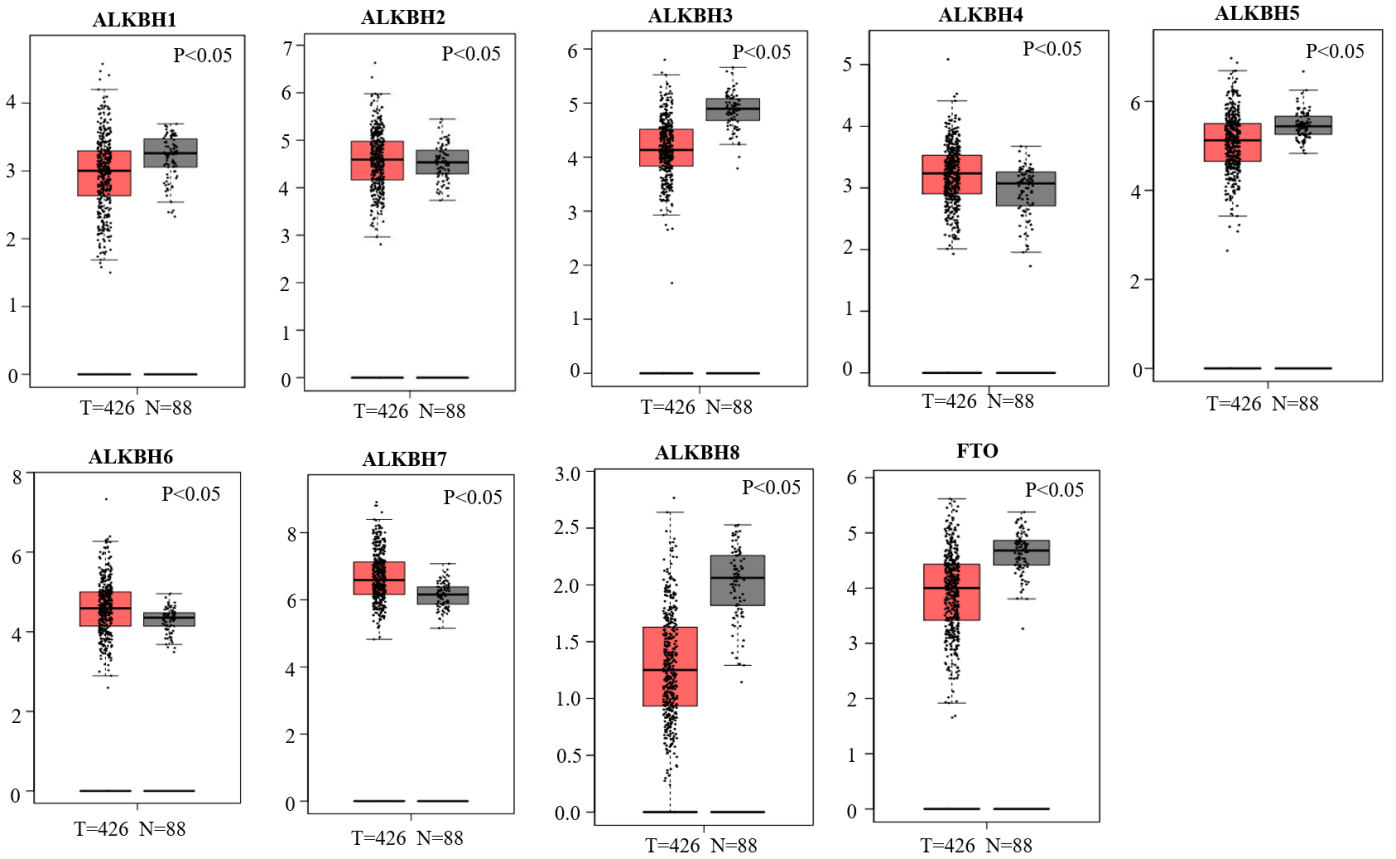
**Differential mRNA expression analysis of the AlkB family in OV and normal tissues.** The expression profiles were collected from the GEPIA databases.

**Figure 2:**
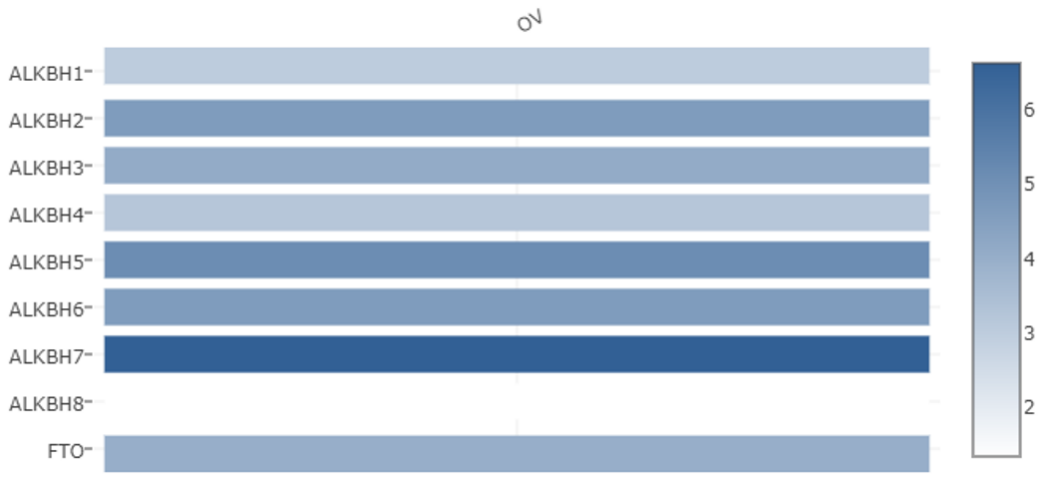
**The relative expression levels of the AlkB family in OV patients.** GEPIA databases were used to evaluate the relative expression levels of the AlkB family in OV patients.

We used the GEPIA databases to evaluate the mRNA expression levels of AlkB family members in OV tissues and normal tissues. We found that the expression levels of ALKBH1, ALKBH3, ALKBH5, ALKBH8 and FTO were significantly downregulated in OV patients. However, the expression levels of ALKBH2, ALKBH4, ALKBH6 and ALKBH7 were higher in OV tissues than in normal tissues (Figure 1). When evaluating the relative levels of the AlkB family in OV tissues, we found that the expression of ALKBH7 was the highest, while that of ALKBH8 was the lowest (Figure 2).

**Figure 3:**
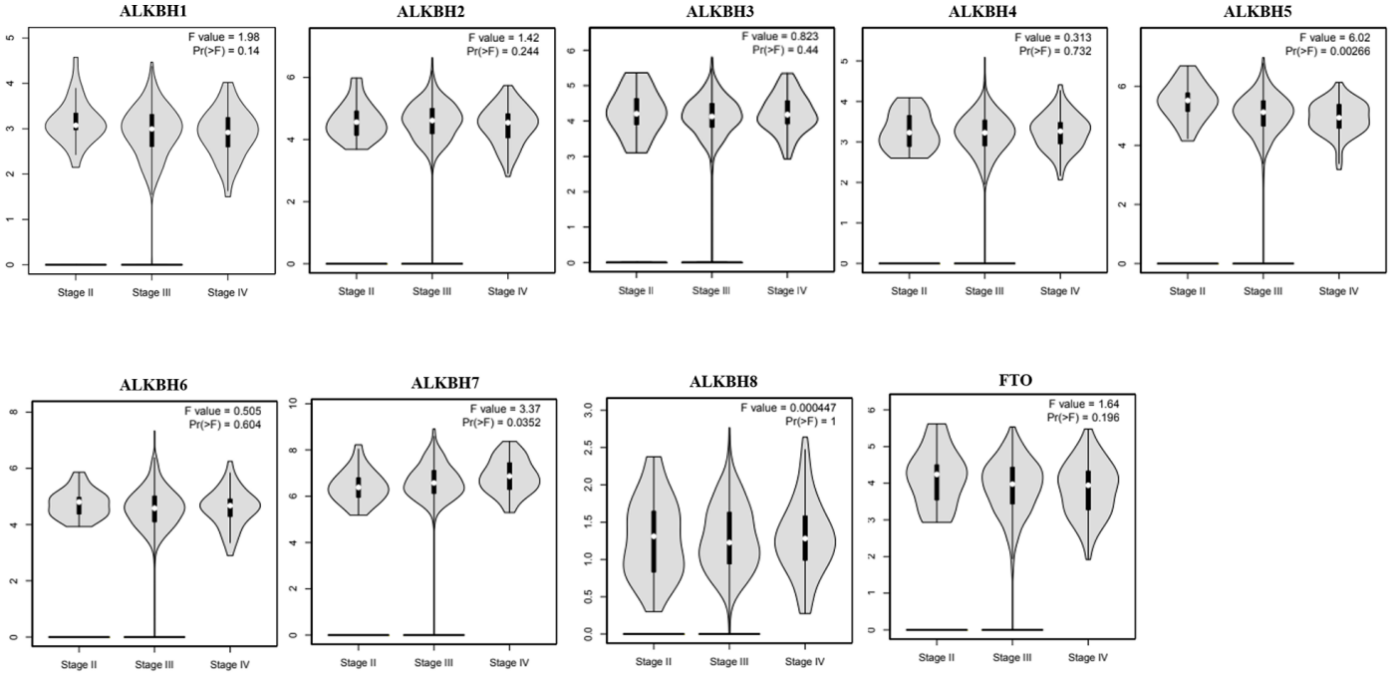
**The relationship between the expression of the AlkB family and the pathological stage of OV patients (GEPIA).** GEPIA databases were used to evaluate the correlations of the AlkB family with the pathological stage of OV patients.

Then, we analyzed the association between the expression profiles of the AlkB family and the pathological stages of OV patients. As shown in Figure 3, ALKBH5 expression (p = 0.00266) displayed a negative correlation with pathological stage. In contrast, ALKBH7 expression (p = 0.0352) displayed a positive correlation with pathological stage. These data suggested that aberrant expression of AlkB family members might participate in disease progression in OV patients.

### The prognostic value of the AlkB family in OV patients

**Figure 4:**
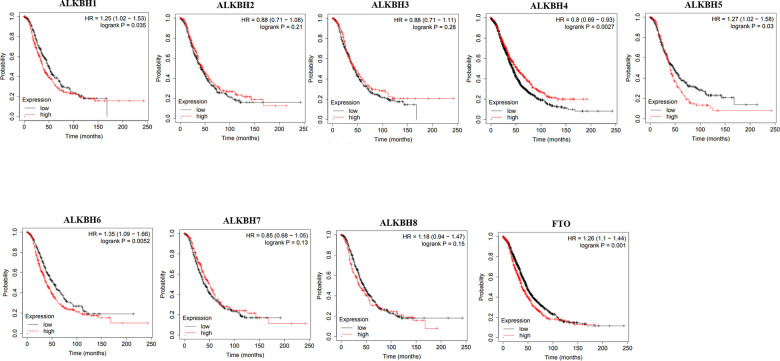
**The correlations of AlkB family expression with patients’ OS.** These OS survival curves were collected from Kaplan-Meier plotter.

**Figure 5:**
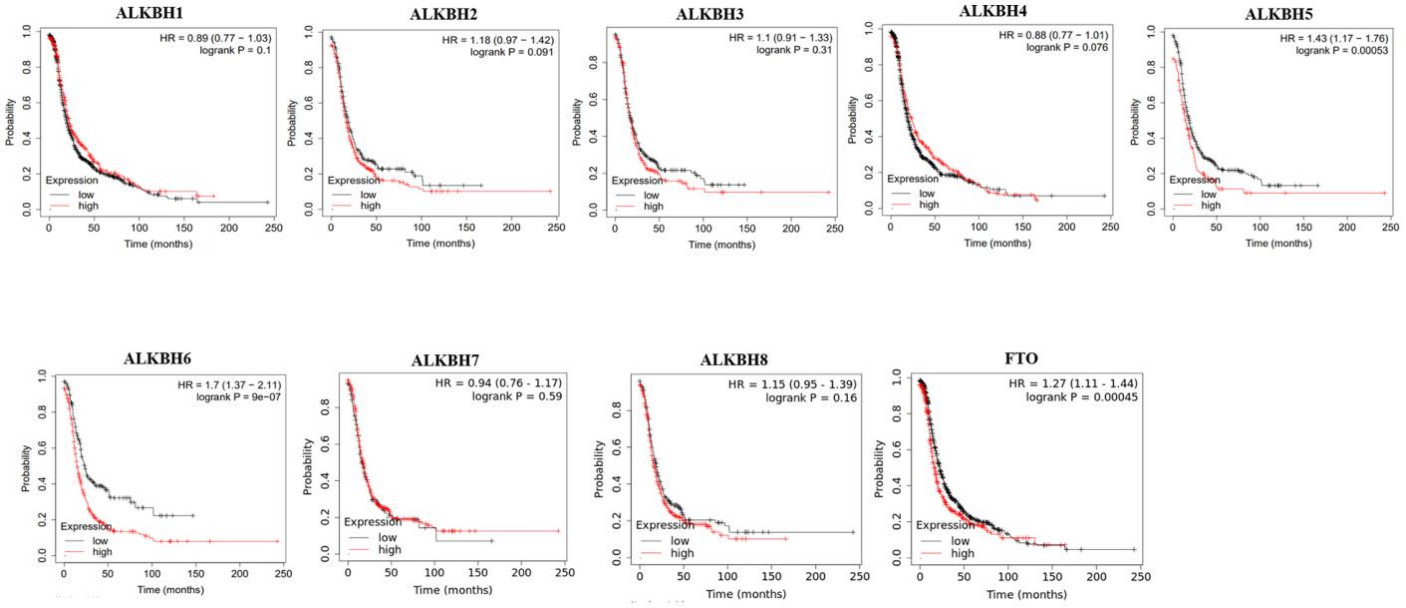
**The correlations of AlkB family expression with PFS in OV patients.** Kaplan-Meier plotter was used to assess the correlation of AlkB family members with the patients’ PFS time.

Then, we used Kaplan-Meier plotter to assess the prognostic value of the AlkB family for patients with OV. The data of the OS curves are shown in Figure 4. We found that a high transcriptional level of ALKBH4 (p = 0.0027) was highly related to a longer OS time. In addition, we also concluded that high transcriptional levels of ALKBH1 (p = 0.035), ALKBH5 (p = 0.03), ALKBH6 (p = 0.0052) and FTO (p = 0.001) had an important association with a shorter OS time. At the same time, we evaluated the prognostic value of the AlkB family on the PFS of OV patients. From Figure 5, we know that low levels of ALKBH5 (p = 0.00053), ALKBH6 (p = 9e-07) and FTO (p = 0.00045) have an important association with a longer PFS time.

### Genetic alteration of the AlkB family in OV patients

**Figure 6:**
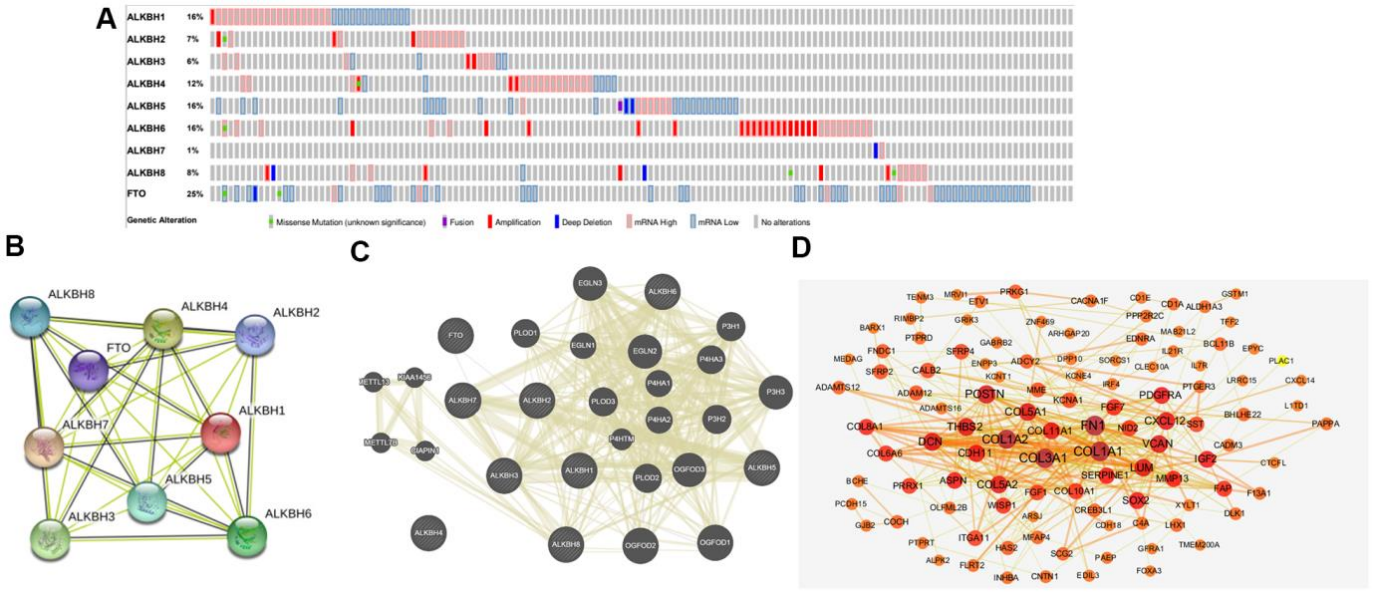
**Genetic alterations and interaction analyses of the AlkB family in OV patients.** (**A**) Genetic alteration of the AlkB family in OV patients analyzed with cBioPortal. (**B**) The interaction analysis of the AlkB family was evaluated by STRING. (**C**) Analysis of the interaction between the AlkB family and chemokine signaling-associated biomarkers. (**D**) The 150 most frequently altered genes identified from cBioPortal that are linked to the AlkB family in OV patients.

To further analyze the alteration profiles of AlkB family members in patients with OV, we conducted a series of surveys as follows. First, we evaluated the genetic alterations of the AlkB family by using the cBioPortal database. As shown in Figure 6A, we confirmed that the alternation rate of FTO was the highest in 25% of cases, whereas the lowest was for ALKBH7, which was only 1%. In addition, ALKBH1, ALKBH2, ALKBH3, ALKBH4, ALKBH5, ALKBH6 and ALKBH8 mutations occurred in 16, 7, 6, 12, 16, 16 and 8% of the samples, respectively (Figure 6A).

### Interaction analyses of the AlkB family in OV patients

Furthermore, we used STRING to evaluate the associated molecules of the AlkB family and found that in the interactive network, 9 AlkB family members were all included and served as hub nodes (Figure 6B). At the same time, to explore the association between the AlkB family and other signaling pathway-associated biomarkers, we used GeneMANIA to find the functions of the AlkB family (ALKBH1, ALKBH2, ALKBH3, ALKBH4, ALKBH5, ALKBH6, ALKBH7, ALKBH8 and FTO) were associated with chemokine signaling pathways, including chemokine receptor binding and cytokine activity (Figure 6C).

Additionally, we used cBioPortal to extract the 150 most frequently altered genes that were significantly linked to the AlkB family in OV patients (Supplementary Table 2). The data indicated that several hub genes, such as COL1A1, COL1A2, COL3A1, FN1, COL5A1 and POSTN, were closely linked to the biological processes of AlkB family modulation in OV patients (Figure 6D).

### Functional enrichment analysis of the AlkB family

**Figure 7:**
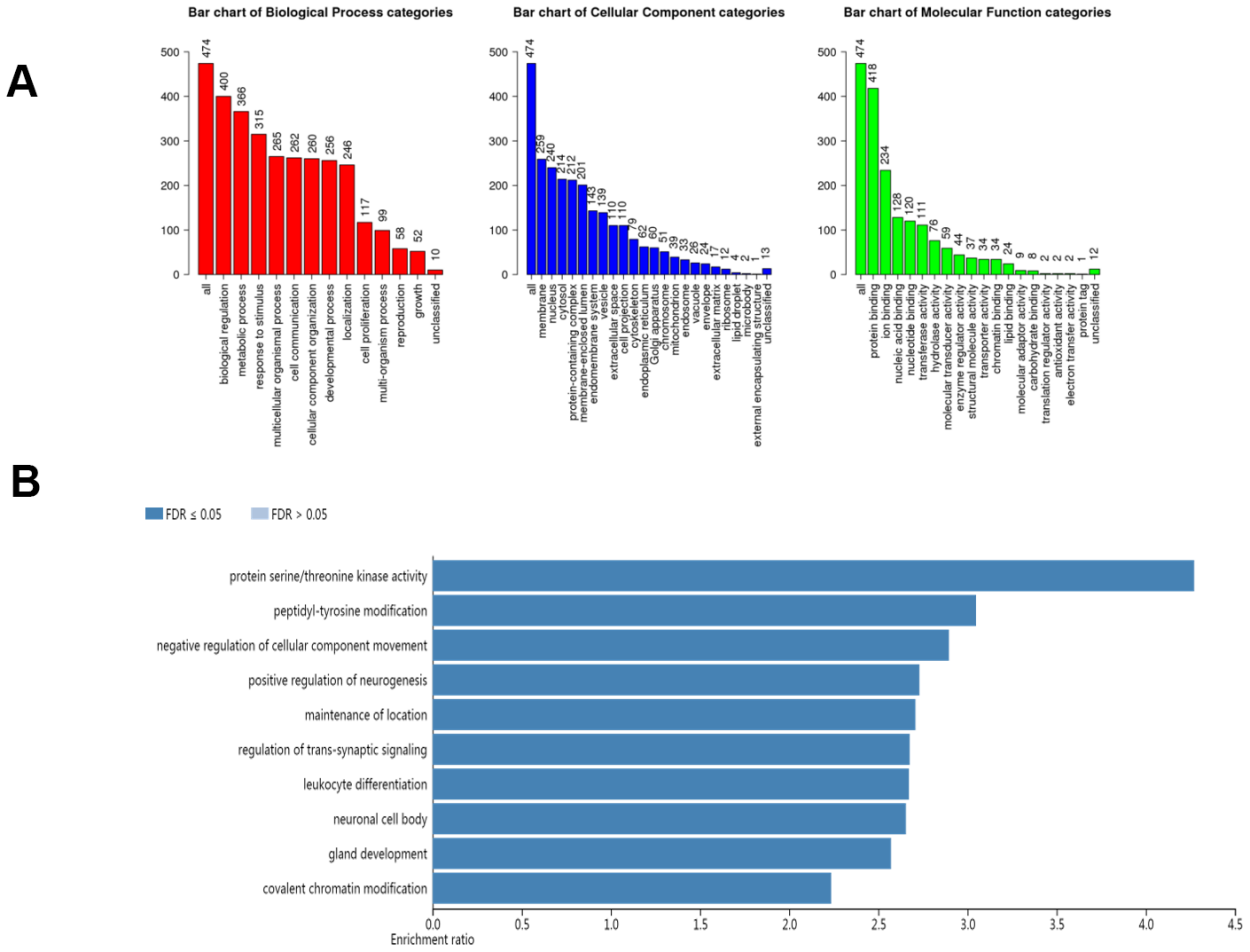
**The biological pathways of the AlkB family were evaluated by the WebGestalt database.** (**A**) Bar plot of GO enrichment in cellular components, biological processes, and molecular functions. (**B**) The bar plot of KEGG enrichment.

In our study, we applied the WebGestalt database to evaluate the biological functions of the AlkB family. As shown in the GO pathways (Figure 7A), we know that the most highly enriched biological process (BP) category was biological regulation, followed by metabolic process, response to stimulus, multicellular organismal process, cell communication, developmental process and localization. In the cellular component (CC) categories, membrane, nucleus, cytosol, protein-containing complex, membrane-enclosed lumen, endomembrane system, vesicle, extracellular space, cell projection, and cytoskeleton were highly enriched. In the molecular function (MF) category, the AlkB family members were mainly enriched in protein binding, ion binding, nucleic acid binding, transferase activity and hydrolase activity. In addition, the KEGG pathway results are shown in Figure 7B. We can conclude from the picture that protein serine/threonine kinase activity, peptidyl-tyrosine modification, negative regulation of cellular component movement, positive regulation of neurogenesis, maintenance of location, regulation of transsynaptic signaling, leukocyte differentiation, neuronal cell body, gland development, gland development and covalent chromatin modification were strongly linked to the potential biological functions of the AlkB family in the occurrence and development of OV.

### Immune cell infiltration of the AlkB family

**Figure 8:**
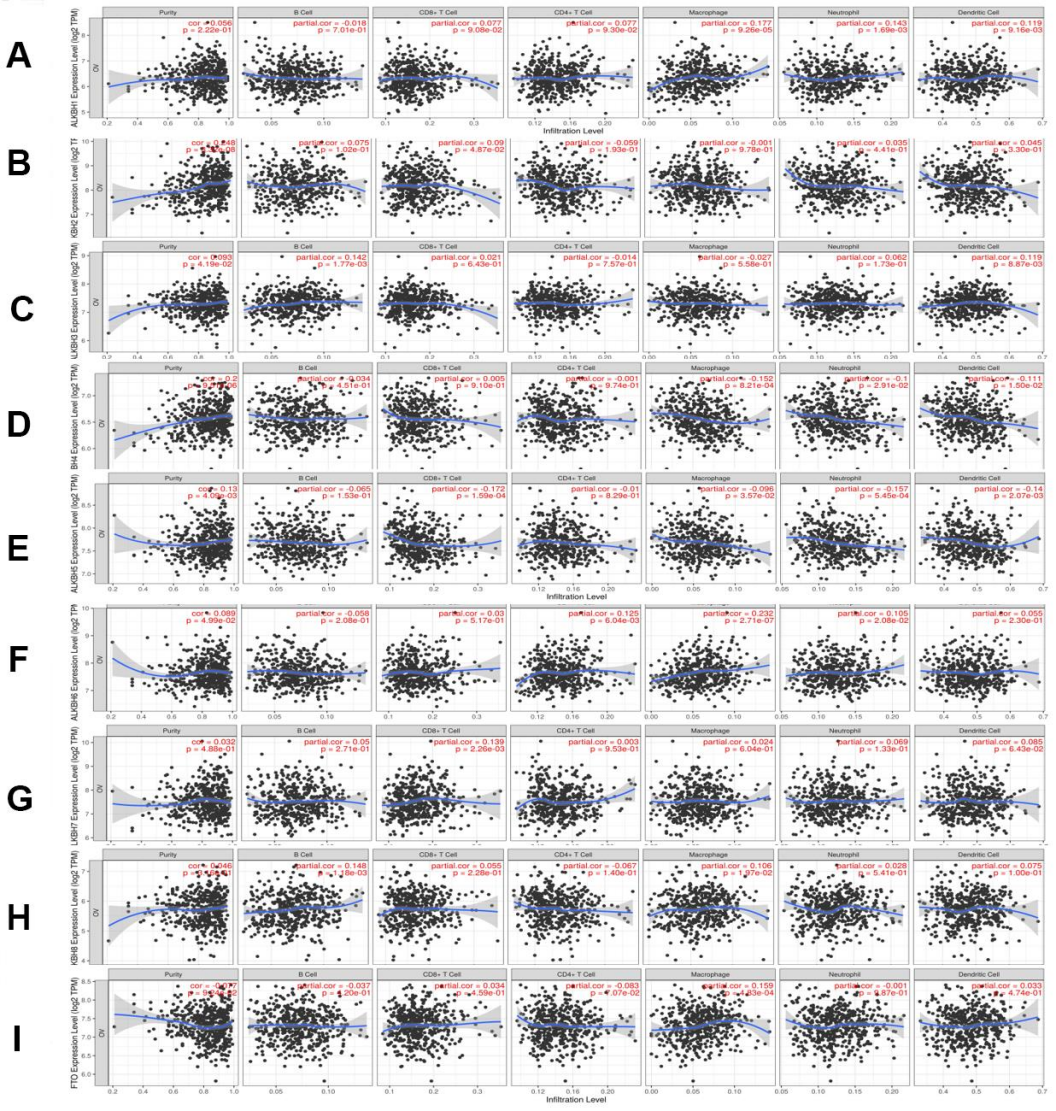
**The relationship between immune cell infiltration and the expression of the AlkB family.** The Timer database was used to analyze the effect of (**A**) ALKBH1, (**B**) ALKBH2, (**C**) ALKBH3, (**D**) ALKBH4, (**E**) ALKBH5, (**F**) ALKBH6, (**G**) ALKBH7, (**H**) ALKBH8 and (**I**) FTO on the abundance of immune cells in OV patients.

To explore the relationship between immune cell infiltration and the expression profiles of the AlkB family, we searched the information from the Timer database. The expression of ALKBH1 was positively linked to the infiltration of macrophages (Cor = 0.177, p = 9.26e-05), neutrophils (Cor =0.143, p = 1.69e-03) and dendritic cells (Cor =0.119, p =9.16e-03) (Figure 8A). ALKBH2 expression had a positive relationship with the infiltration of CD8+ T cells (Cor = 0.09, p = 4.87e-02) and B cells (Cor = 0.075, p = 1.02e-01), whereas it had a negative relationship with the infiltration of CD4+ T cells (Cor = -0.059, p = 1.93e-01) (Figure 8B). There was a positive correlation between ALKBH3 expression and the infiltration of B cells (Cor = 0.142, p = 1.77e-03), dendritic cells (Cor = 0.119, p = 8.87e-03) and neutrophils (Cor = 0.062, p = 1.73e-01) (Figure 8C). We concluded from Figure 8D that ALKBH4 expression was negatively associated with the infiltration of macrophages (Cor = -0.152, p = 8.21e-04), dendritic cells (Cor = -0.111, p = 1.50e-02) and neutrophils (Cor = -0.1, p = 2.91e-02). There was a negative correlation between ALKBH5 expression and the infiltration of CD8+ T cells (Cor = -0.172, p = 1.59e-04), neutrophils (Cor = -0.157, p = 5.45e-04) and dendritic cells (Cor = -0.14, p = 2.07e-03) (Figure 8E). We also found that the expression of ALKBH6 was positively related to the infiltration of macrophages (Cor = 0.232, p = 2.71e-07), CD4+ T cells (Cor = 0.125, p = 6.04e-03) and neutrophils (Cor = 0.105, p = 2.08e-02) (Figure 8F). There was a positive correlation between ALKBH7 expression and the infiltration of CD8+ T cells (Cor = 0.139, p = 2.26e-03), dendritic cells (Cor = 0.085, p = 6.43e-02) and neutrophils (Cor = 0.069, p = 1.33e-01) (Figure 8G). There was a positive correlation between ALKBH8 expression and the infiltration of B cells (Cor = 0.148, p = 1.18e-03) and macrophages (Cor = 0.106, p = 1.97e-02) (Figure 8H). Additionally, the expression of FTO was positively linked to the infiltration of macrophages (Cor = 0.159, p = 4.83e-04) and negatively linked to the infiltration of CD4+ T cells (Cor = -0.083, p = 7.07e-02) (Figure 8I). At the same time, we analyzed the relationship between the expression of the AlkB family and the infiltration of immune cells (Table 1). The Cox proportional hazard model included the following confounding factors: B cells, CD8+ T cells, CD4+ T cells, macrophages, neutrophils, dendritics, ALKBH1, ALKBH2, ALKBH3, ALKBH4, ALKBH5, ALKBH6, ALKBH7, ALKBH8 and FTO. From Table 1, we found that CD4+ T cells (p = 0.000) and macrophages (p = 0.007) had a strong relationship with OV patient prognosis.

**Table 1:** The cox proportional hazard model of AlkB family and six types of immune cells in OV patients from timer database.

	**Coef**	**HR**	**95%CI_l**	**95%CI_u**	**P.value**	**Sig**
B_cell	-0.940	0.391	0.000	3.103130e+02	0.783	
CD8_Tcell	-3.190	0.041	0.001	2.219000e+00	0.117	
CD4_Tcell	-18.637	0.000	0.000	0.000000e+00	0.000	***
Macrophage	8.116	3346.999	9.360	1.196836e+06	0.007	**
Neutrophil	11.984	160162.297	13.497	1.900588e+09	0.012	*
Dendritic	-3.508	0.030	0.000	5.193000e+00	0.182	
ALKBH1	0.001	1.001	0.787	1.272000e+00	0.996	
ALKBH2	-0.079	0.924	0.751	1.137000e+00	0.457	
ALKBH3	-0.154	0.857	0.638	1.152000e+00	0.307	
ALKBH4	-0.289	0.749	0.492	1.139000e+00	0.177	
ALKBH5	-0.327	0.721	0.513	1.014000e+00	0.060	
ALKBH6	0.043	1.044	0.803	1.358000e+00	0.749	
ALKBH7	-0.102	0.903	0.745	1.095000e+00	0.299	
ALKBH8	0.117	1.125	0.918	1.377000e+00	0.256	
FTO	0.175	1.191	0.895	1.584000e+00	0.230	

### Methylation level of the AlkB family

**Figure 9:**
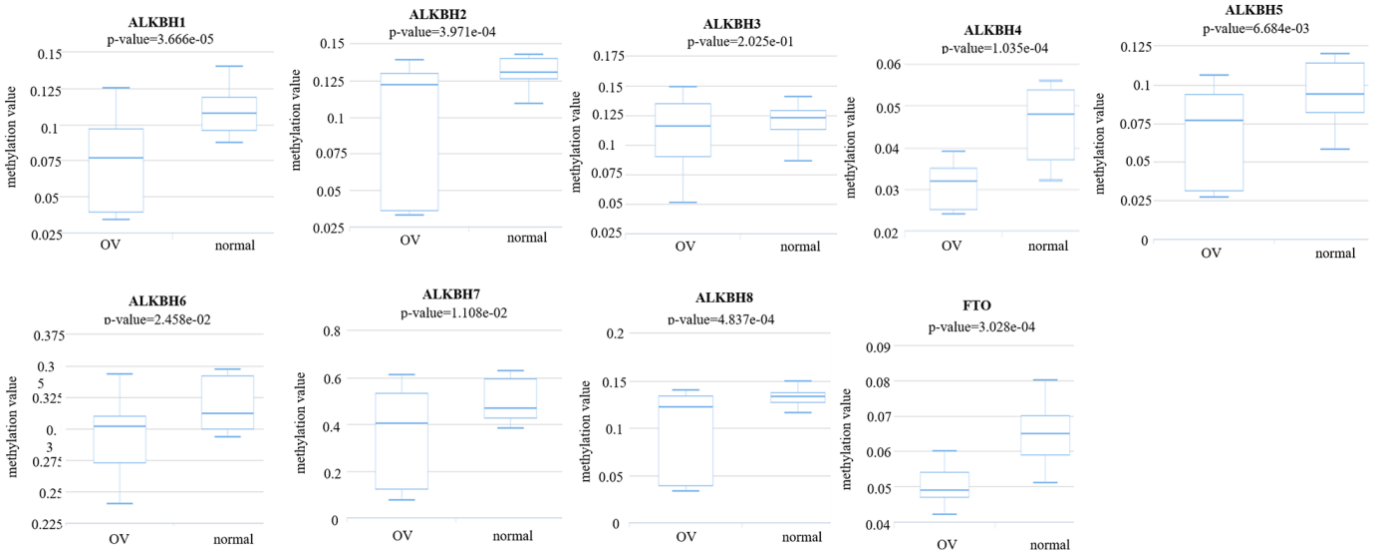
**The methylation levels of the AlkB family in OV tissues.** The methylation values of AlkB family members were evaluated using the DiseaseMeth database.

Then, we analyzed the methylation levels of the AlkB family by searching the DiseaseMeth database. We found that the methylation levels of ALKBH1 (p = 3.666e-05), ALKBH2 (p = 3.971e-04), ALKBH3 (p = 2.025e-01), ALKBH4 (p = 1.035e-04), ALKBH5 (p = 6.684e-03), ALKBH6 (p = 2.458e-02), ALKBH7 (p = 1.108e-02), ALKBH8 (p = 4.837e-04) and FTO (p = 3.028e-04) were all lower in OV patients than in healthy people (Figure 9). These downregulated methylation values might explain their difference in expression levels to some extent.

## DISCUSSION

AlkB family members have recently been identified to be involved in the regulation of RNA m6A modification. AlkB family members mainly function as demethylases [20]. Some reports have shown that ALKBH5 (ALKB homolog 5) has a strong correlation with the inhibition of cancer growth [21]. A study indicated that ALKBH2 is highly expressed in bladder cancer [22]. Furthermore, two research groups have demonstrated that ALKBH1 and ALKBH5 are related to glioblastoma [23, 24]. At the same time, ALKBH5 could serve as a tumor suppressor in the proliferation inhibition of HCC cells [25]. Previous study has shown that meclofenamic acid (MA) could serve as FTO inhibitor, which provided future directions of applying FTO inhibitors in the medicine [26]. Additionally, a report demonstrated that pharmacological function of FTO inhibitor could slow down the self-renewal of leukemia stem cells [27]. However, the detailed functions and mechanisms of the AlkB family in OV have not been fully explored and explained.

In addition, a recent study demonstrated that the high expression of ALKBH5 played a significant role in the proliferation and invasion of OV cells [19]. Moreover, from the GEPIA database, we also concluded that the expression of the AlkB family is different in OV and normal tissues (upregulation of ALKBH2, ALKBH4, ALKBH6 and ALKBH7; downregulation of ALKBH1, ALKBH3, ALKBH5, ALKBH8 and FTO). Additionally, we also performed a correlation between the AlkB family and the patients’ pathological stage. We found that the expression of ALKBH5 decreased as OV cancer progressed and that the expression of ALKBH7 increased as OV cancer progressed. OV patients with low levels of ALKBH1, ALKBH5, ALKBH6 and FTO had an association with longer OS curves. OV patients with low levels of ALKBH5, ALKBH6 and FTO had an association with better PFS curves. The results of these databases implied that the expression of the AlkB family is crucial for the progression of OV patients. However, more specific studies are needed to explore the expression and prognostic value of the AlkB family in OV patients.

We explored the biological functions of the AlkB family by analyzing the GO and the KEGG pathways. These two enrichment pathways are targeted at exploring the functional meanings of genes at the molecular level and the biological interpretation of genome sequences [28–30]. Through this study, we found that the biological functions of the AlkB family were mainly linked to posttranslational modification pathways, such as protein serine/threonine kinase activity and peptidyl-tyrosine modification. A previous study has shown that tyrosine modification plays a significant role in the regulation of gene expression and the therapeutic response of ovarian cancer [31]. Accordingly, from other previous studies, enrichment pathway analysis revealed that protein serine/threonine kinase activity could also participate in the therapeutic response of epithelial ovarian cancer [32, 33]. Therefore, we can conclude that the AlkB family may be involved in the progression of OV by regulating protein posttranslational modification pathways.

At present, there are many studies that indicate that RNA m6A methylation could modulate immunity, regulate tumor inflammation, and predict patient outcomes [34, 35]. In addition, some studies have revealed that immune cells could have important effects on the treatment efficacy of human cancers, such as in clinical immunotherapy [36, 37]. In our study, we found that the expression of the AlkB family is significantly linked to the infiltration of CD4+ T cells, macrophages and neutrophils. These data indicated that the AlkB family could regulate the infiltration of immune cells, which may affect the immune response of infiltrating immune cells in the tumor environment.

However, our study still has some limitations. Firstly, although the study demonstrated that molecular profiles of the AlkB family would be a potential indicator of OV, the statistics we analyzed were all from the databases, lacking experimental researches. Secondly, the study did not conduct some survey concerning the therapeutic outcome of OV patients, we still need further studies to validate the therapeutic significance of the AlkB family. Thirdly, because of inconsistent data-sets used by the databases, some contradictory data need to be further clarified. Finally, it is important to explore more prognostic effects of OV patients in order to improve the application value of the AlkB family.

In conclusion, our study evaluated the molecular profiles of the AlkB family in patients with OV. Moreover, the findings in this study might also be beneficial for the development of better diagnostic and treatment methods for OV patients to improve their prognosis.

## MATERIALS AND METHODS

### GEPIA

Gene Expression Profiling Interactive Analysis (GEPIA) is a database that is designed to help end users fully understand gene expression from a more holistic perspective [38]. We used “Single Gene Analysis” in GEPIA to evaluate the expression profiles of AlkB family members in OV tissues compared with normal tissues based on the data from TCGA and GTEx. We also used this database to evaluate the roles of the AlkB family in pathological stage, prognosis and so on. We set p-values at 0.05.

### Kaplan-Meier plotter

Kaplan-Meier plotter is a database assessing the relationship between gene expression and the survival trend of various cancer patients [39]. In our study, we evaluated the prognosis of OV patients by means of overall survival (OS) and progression-free survival (PFS) curves. Additionally, we could identify the high expression and low expression groups from the figures, and it showed statistical significance if the p-value < 0.05.

### cBioPortal

cBioPortal includes statistics on over 200 cancer genomics and provides a user-friendly analysis strategy concerning gene-disease associations [40]. In this study, we analyzed the coexpression profiles and genetic alterations of the AlkB family in OV tissues by searching cBioPortal.

### STRING

STRING was applied to explore potential protein-protein interactions (PPIs). Furthermore, this database provides all resources concerning the interactive network of multiple proteins [41]. At the same time, we analyzed the AlkB family member-associated PPI network using STRING and Cytoscape [42].

### GeneMANIA

GeneMANIA has been applied in scientific research for many years and is very convenient for identifying protein-protein interactive networks [43]. Using GeneMANIA, we successfully identified the genes associated with the AlkB family.

### WebGestalt

WebGestalt aims to provide users with a better understanding of gene interpretation. Researchers can obtain enrichment results from this database [44]. In our study, we analyzed several enrichment pathways associated with the AlkB family in OV disease. The main two enrichment pathways are Gene Ontology (GO) and Kyoto Encyclopedia of Genes and Genomes (KEGG).

### Timer

Timer is used to evaluate the connection between infiltrating immune cells and cancer cells, which could provide more rational strategies for an improved therapeutic response and prognosis [45]. We mainly performed correlation analysis between OV and immune cells using the Timer database.

### DiseaseMeth

DiseaseMeth collects the database of DNA methylation, which has a strong relationship with gene expression and disease incidence [46]. In our study, we evaluated the relationship between the expression of the AlkB family and methylation levels. We also analyzed whether methylation levels of the AlkB family have a potential influence on the survival of OV patients.

## Supplementary Material

Supplementary Table 1

Supplementary Table 2
