# Supplementary material for: Expression and molecular profiles of the AlkB family in ovarian serous carcinoma
Source: Aging (Albany NY). 2021 Mar 19;13(7):9679–92. doi: 10.18632/aging.202716 (PMC8064172; doi:10.18632/aging.202716)
Supplement: Supplementary Table 1 [file aging-13-202716-s001.pdf]

Supplementary Table 1. The main databases applied to evaluate the expression and functions of AlkB family in the biological process of ovarian serous carcinoma.

Databases	Authors	Publication date	Samples	URL
GEPIA2	Tang Z. et al.	2019	Tissues	http://gepia.cancer-pku.cn/
Kaplan-Meier plotter	Gyorffy B. et al.	2005	Tissues	http://kmplot.com/analysis/
cBioPortal	Cerami E. et al.	2012	Tissues	http://www.cbioportal.org/
STRING v11	Szklarczyk D. et al.	2019	-	https://string-db.org/
GeneMANIA	Warde-Farley D. et al.	2010	-	http://genemania.org/
WebGestalt	Liao Y. et al.	2019	-	http://webgestalt.org/
Timer2.0	Li T. et al.	2020	Tissues	https://cistrome.shinyapps.io/timer/
DiseaseMeth2.0	Xiong Y. et al.	2017	Tissues	http://bio-bigdata.hrbmu.edu.cn/diseasemeth/

GEPIA: Gene expression profiling interactive analysis; WebGestalt: the web-based GEne SeT AnaLysis Toolkit.
