# Supplementary material for: Expression and molecular profiles of the AlkB family in ovarian serous carcinoma
Source: Aging (Albany NY). 2021 Mar 19;13(7):9679–92. doi: 10.18632/aging.202716 (PMC8064172; doi:10.18632/aging.202716)
Supplement: Supplementary Table 2 [file aging-13-202716-s002.doc]

**Supplementary Table 2. The database of cBioportal applied to search the altered genes of the AlkB family in tissues of ovarian serous carcinoma.**

| **Gene** | **Cytoband** | **Log Ratio** | **p-Value** | **q-Value** | **Expression** |
| --- | --- | --- | --- | --- | --- |
| MMP13 | 11q22.2 | -1.38 | 8.79E-03 | 0.12 | Unaltered group |
| GRIK3 | 1p34.3 | -1.36 | 3.08E-03 | 0.0744 | Unaltered group |
| SORCS1 | 10q25.1 | -1.29 | 2.22E-03 | 0.0648 | Unaltered group |
| PTPRT | 20q12-q13.11 | -1.28 | 7.81E-03 | 0.114 | Unaltered group |
| CNTN1 | 12q12 | -1.27 | 1.12E-05 | 0.0138 | Unaltered group |
| PIEZO2 | 18p11.22-p11.21 | -1.27 | 1.16E-05 | 0.0138 | Unaltered group |
| GSTM1 | 1p13.3 | -1.21 | 0.0616 | 0.296 | Unaltered group |
| TENM3 | 4q34.3-q35.1 | -1.2 | 3.13E-03 | 0.0751 | Unaltered group |
| ADAMTS16 | 5p15.32 | -1.19 | 8.24E-04 | 0.0441 | Unaltered group |
| EPYC | 12q21.33 | -1.15 | 0.0244 | 0.188 | Unaltered group |
| COL8A1 | 3q12.1 | -1.14 | 2.23E-04 | 0.0279 | Unaltered group |
| SVEP1 | 9q31.3 | -1.12 | 4.72E-04 | 0.0362 | Unaltered group |
| COL11A1 | 1p21.1 | -1.12 | 0.0279 | 0.203 | Unaltered group |
| LUM | 12q21.33 | -1.11 | 1.04E-03 | 0.0477 | Unaltered group |
| FAP | 2q24.2 | -1.11 | 4.15E-03 | 0.0859 | Unaltered group |
| NTM | 11q25 | -1.11 | 5.00E-03 | 0.0926 | Unaltered group |
| COL6A6 | 3q22.1 | -1.1 | 3.85E-03 | 0.0836 | Altered group |
| PTGER3 | 1p31.1 | -1.09 | 1.13E-03 | 0.0485 | Altered group |
| BCHE | 3q26.1 | -1.06 | 4.44E-03 | 0.088 | Altered group |
| SOX2 | 3q26.33 | -1.06 | 0.0242 | 0.188 | Unaltered group |
| SFRP2 | 4q31.3 | -1.06 | 0.0447 | 0.253 | Altered group |
| ADAM12 | 10q26.2 | -1.05 | 5.93E-04 | 0.0396 | Unaltered group |
| LRRC15 | 3q29 | -1.05 | 0.0191 | 0.168 | Unaltered group |
| CDH18 | 5p14.3 | -1.05 | 0.0209 | 0.176 | Unaltered group |
| BICC1 | 10q21.1 | -1.04 | 4.11E-05 | 0.0164 | Unaltered group |
| VGLL3 | 3p12.1 | -1.04 | 1.54E-04 | 0.025 | Altered group |
| COL3A1 | 2q32.2 | -1.03 | 3.97E-04 | 0.0349 | Unaltered group |
| IL7R | 5p13.2 | -1.03 | 6.30E-04 | 0.0404 | Unaltered group |
| VCAN | 5q14.2-q14.3 | -1.02 | 6.34E-04 | 0.0406 | Unaltered group |
| SERPINB7 | 18q21.33 | -1.02 | 0.011 | 0.132 | Unaltered group |
| IGF2 | 11p15.5 | -1.02 | 0.0479 | 0.263 | Unaltered group |
| FNDC1 | 6q25.3 | -1.01 | 1.74E-04 | 0.0259 | Unaltered group |
| THBS2 | 6q27 | -1.01 | 1.29E-03 | 0.051 | Unaltered group |
| MME | 3q25.2 | -1.01 | 1.81E-03 | 0.0581 | Unaltered group |
| DPP10 | 2q14.1 | -1.01 | 0.0136 | 0.146 | Unaltered group |
| ADAM6 | 14q32.33 | -1.01 | 0.0401 | 0.24 | Unaltered group |
| CRISPLD2 | 16q24.1 | -1 | 1.09E-04 | 0.022 | Unaltered group |
| VAT1L | 16q23.1 | -1 | 1.28E-04 | 0.023 | Unaltered group |
| LRRN4 | 20p12.3 | -1 | 3.70E-04 | 0.0341 | Unaltered group |
| PAPPA | 9q33.1 | -1 | 4.15E-04 | 0.0353 | Unaltered group |
| AQP9 | 15q21.3 | -1 | 1.14E-03 | 0.0485 | Unaltered group |
| ALPK2 | 18q21.31-q21.32 | -1 | 3.78E-03 | 0.0829 | Altered group |
| CLDN16 | 3q28 | -0.99 | 0.0214 | 0.178 | Unaltered group |
| LHX1 | 17q12 | -0.99 | 0.0592 | 0.291 | Unaltered group |
| NCAM2 | 21q21.1 | -0.98 | 1.76E-03 | 0.0572 | Altered group |
| SEMA3D | 7q21.11 | -0.98 | 4.27E-03 | 0.0871 | Unaltered group |
| GALNT5 | 2q24.1 | -0.97 | 4.95E-04 | 0.0363 | Unaltered group |
| CD1A | 1q23.1 | -0.97 | 0.0106 | 0.13 | Unaltered group |
| LSAMP | 3q13.31 | -0.96 | 7.14E-04 | 0.0416 | Unaltered group |
| COL5A1 | 9q34.3 | -0.96 | 1.11E-03 | 0.0483 | Unaltered group |
| COL1A1 | 17q21.33 | -0.95 | 9.02E-04 | 0.0451 | Unaltered group |
| KCNA1 | 12p13.32 | -0.94 | 2.91E-04 | 0.0308 | Altered group |
| ADAMTS12 | 5p13.3-p13.2 | -0.94 | 9.34E-04 | 0.0457 | Unaltered group |
| CXCL14 | 5q31.1 | -0.94 | 0.0167 | 0.159 | Unaltered group |
| POSTN | 13q13.3 | -0.94 | 0.048 | 0.263 | Altered group |
| ITGA11 | 15q23 | -0.93 | 1.30E-03 | 0.051 | Unaltered group |
| GABRB2 | 5q34 | -0.93 | 1.55E-03 | 0.0549 | Unaltered group |
| COL10A1 | 6q22.1 | -0.92 | 0.0162 | 0.157 | Unaltered group |
| ZNF469 | 16q24.2 | -0.91 | 6.94E-05 | 0.019 | Unaltered group |
| FGF7 | 15q21.2 | -0.91 | 1.26E-03 | 0.0502 | Unaltered group |
| SERPINE1 | 7q22.1 | -0.91 | 2.03E-03 | 0.0619 | Unaltered group |
| IGFL2 | 19q13.32 | -0.91 | 0.0216 | 0.178 | Unaltered group |
| PLPPR4 | 1p21.3-p21.2 | -0.9 | 2.09E-04 | 0.0274 | Unaltered group |
| ZFHX4 | 8q21.13 | -0.9 | 4.21E-04 | 0.0353 | Altered group |
| FIBCD1 | 9q34.12 | -0.9 | 5.62E-04 | 0.0384 | Unaltered group |
| FN1 | 2q35 | -0.9 | 2.15E-03 | 0.0639 | Unaltered group |
| SLC22A3 | 6q25.3 | -0.9 | 2.22E-03 | 0.0648 | Altered group |
| DIO2 | 14q31.1 | -0.9 | 0.0147 | 0.151 | Unaltered group |
| H19 | 11p15.5 | -0.9 | 0.0652 | 0.305 | Unaltered group |
| CCN4 | 8q24.22 | -0.89 | 9.55E-05 | 0.0214 | Altered group |
| CXCL12 | 10q11.21 | -0.89 | 6.22E-04 | 0.0404 | Unaltered group |
| CLEC10A | 17p13.1 | -0.89 | 8.58E-04 | 0.0448 | Unaltered group |
| HAS2 | 8q24.13 | -0.89 | 1.51E-03 | 0.0542 | Altered group |
| MAB21L2 | 4q31.3 | -0.89 | 2.06E-03 | 0.0623 | Unaltered group |
| CD1E | 1q23.1 | -0.89 | 5.28E-03 | 0.0954 | Altered group |
| INHBA | 7p14.1 | -0.89 | 0.0102 | 0.128 | Altered group |
| XYLT1 | 16p12.3 | -0.88 | 3.61E-05 | 0.0164 | Unaltered group |
| ARSJ | 4q26 | -0.88 | 4.06E-05 | 0.0164 | Unaltered group |
| F13A1 | 6p25.1 | -0.88 | 6.71E-05 | 0.019 | Unaltered group |
| KCNE4 | 2q36.1 | -0.88 | 6.50E-04 | 0.0407 | Unaltered group |
| RUNX1T1 | 8q21.3 | -0.88 | 1.30E-03 | 0.051 | Unaltered group |
| PRRX1 | 1q24.2 | -0.88 | 4.20E-03 | 0.0863 | Altered group |
| CALB2 | 16q22.2 | -0.88 | 0.0168 | 0.159 | Unaltered group |
| CADM3 | 1q23.2 | -0.88 | 0.0713 | 0.318 | Altered group |
| CSMD2 | 1p35.1 | -0.87 | 4.41E-03 | 0.0879 | Unaltered group |
| GFRA1 | 10q25.3 | -0.87 | 0.0136 | 0.146 | Unaltered group |
| ADCY2 | 5p15.31 | -0.87 | 0.0181 | 0.164 | Unaltered group |
| OLFML2B | 1q23.3 | -0.86 | 1.12E-04 | 0.0221 | Unaltered group |
| MEDAG | 13q12.3 | -0.86 | 1.74E-03 | 0.0572 | Unaltered group |
| COL5A2 | 2q32.2 | -0.86 | 1.96E-03 | 0.0609 | Unaltered group |
| MFAP4 | 17p11.2 | -0.86 | 4.98E-03 | 0.0923 | Altered group |
| SCG2 | 2q36.1 | -0.86 | 5.83E-03 | 0.0992 | Unaltered group |
| PRKG1 | 10q11.23-q21.1 | -0.85 | 4.71E-05 | 0.0164 | Altered group |
| ALDH1A3 | 15q26.3 | -0.85 | 7.59E-04 | 0.0421 | Unaltered group |
| BHLHE22 | 8q12.3 | -0.85 | 1.75E-03 | 0.0572 | Unaltered group |
| GJB2 | 13q12.11 | -0.85 | 0.0129 | 0.141 | Unaltered group |
| BCL11B | 14q32.2 | -0.84 | 2.78E-04 | 0.0303 | Unaltered group |
| PDGFRA | 4q12 | -0.84 | 1.42E-03 | 0.0533 | Altered group |
| IRF4 | 6p25.3 | -0.84 | 7.54E-03 | 0.111 | Altered group |
| RIMBP2 | 12q24.33 | -0.84 | 9.69E-03 | 0.125 | Altered group |
| MRVI1 | 11p15.4 | -0.83 | 7.80E-06 | 0.0138 | Unaltered group |
| NID2 | 14q22.1 | -0.83 | 2.10E-05 | 0.0138 | Altered group |
| FAM180A | 7q33 | -0.83 | 3.66E-04 | 0.0339 | Altered group |
| COL1A2 | 7q21.3 | -0.83 | 4.91E-04 | 0.0362 | Altered group |
| DCN | 12q21.33 | -0.83 | 1.73E-03 | 0.0572 | Unaltered group |
| L1TD1 | 1p31.3 | -0.83 | 5.86E-03 | 0.0992 | Unaltered group |
| BARX1 | 9q22.32 | -0.83 | 0.0256 | 0.194 | Unaltered group |
| ESRG | 3p14.3|3p14.3 | -0.83 | 0.0379 | 0.234 | Altered group |
| FLRT2 | 14q31.3 | -0.82 | 5.70E-05 | 0.0183 | Altered group |
| ETV1 | 7p21.2 | -0.82 | 9.95E-05 | 0.0214 | Altered group |
| SLC38A5 | Xp11.23 | -0.82 | 2.29E-03 | 0.0659 | Unaltered group |
| IL21R | 16p12.1 | -0.82 | 4.92E-03 | 0.0922 | Altered group |
| ASPN | 9q22.31 | -0.82 | 0.0135 | 0.145 | Altered group |
| TENM3-AS1 | 4q34.3 | -0.82 | 0.0152 | 0.153 | Unaltered group |
| SFRP4 | 7p14.1 | -0.82 | 0.0156 | 0.154 | Unaltered group |
| CREB3L1 | 11p11.2 | -0.81 | 2.33E-04 | 0.0284 | Altered group |
| EDIL3 | 5q14.3 | -0.81 | 6.13E-04 | 0.0401 | Unaltered group |
| C4A | 6p21.33 | -0.81 | 4.06E-03 | 0.0851 | Altered group |
| PTPRD | 9p24.1-p23 | -0.81 | 7.67E-03 | 0.112 | Unaltered group |
| ABCA8 | 17q24.2 | -0.81 | 8.51E-03 | 0.118 | Unaltered group |
| EDNRA | 4q31.22-q31.23 | -0.8 | 4.19E-05 | 0.0164 | Unaltered group |
| TMEM200A | 6q23.1 | -0.8 | 7.11E-05 | 0.019 | Altered group |
| FGF1 | 5q31.3 | -0.8 | 4.05E-04 | 0.0349 | Unaltered group |
| ARHGAP20 | 11q22.3-q23.1 | -0.8 | 7.67E-04 | 0.0423 | Altered group |
| CDH11 | 16q21 | -0.8 | 1.04E-03 | 0.0477 | Altered group |
| PCDHGA12 | 5q31.3 | -0.8 | 1.58E-03 | 0.0553 | Unaltered group |
| COL6A5 | 3q22.1 | -0.8 | 5.54E-03 | 0.0967 | Unaltered group |
| PAEP | 9q34.3 | -0.8 | 0.0869 | 0.351 | Altered group |
| DLL3 | 19q13.2 | 0.8 | 3.34E-03 | 0.0779 | Unaltered group |
| PPP2R2C | 4p16.1 | 0.8 | 0.0559 | 0.284 | Unaltered group |
| ITIH6 | Xp11.22 | 0.81 | 4.22E-04 | 0.0353 | Unaltered group |
| ENPP3 | 6q23.2 | 0.81 | 0.0114 | 0.133 | Unaltered group |
| PPM1N | 19q13.32 | 0.82 | 4.72E-04 | 0.0362 | Unaltered group |
| PCDH15 | 10q21.1 | 0.82 | 1.76E-03 | 0.0572 | Unaltered group |
| ALG1L | 3q21.2 | 0.85 | 3.95E-03 | 0.084 | Altered group |
| FSTL4 | 5q31.1 | 0.86 | 0.012 | 0.137 | Unaltered group |
| DLK1 | 14q32.2 | 0.86 | 0.115 | 0.399 | Unaltered group |
| COCH | 14q12 | 0.87 | 5.93E-03 | 0.1 | Unaltered group |
| EXOC3L4 | 14q32.32 | 0.88 | 6.80E-04 | 0.0413 | Unaltered group |
| SULT2B1 | 19q13.33 | 0.89 | 2.23E-03 | 0.0648 | Unaltered group |
| AZGP1 | 7q22.1 | 0.91 | 0.0166 | 0.159 | Unaltered group |
| PNMT | 17q12 | 0.92 | 8.78E-04 | 0.045 | Unaltered group |
| KCNT1 | 9q34.3 | 0.93 | 0.0152 | 0.153 | Unaltered group |
| FOXA3 | 19q13.32 | 0.94 | 7.84E-03 | 0.114 | Altered group |
| TFF2 | 21q22.3 | 0.97 | 8.02E-05 | 0.0203 | Unaltered group |
| PPP1R14D | 15q15.1 | 0.97 | 1.16E-04 | 0.0223 | Altered group |
| CNTD2 | 19q13.2 | 1.02 | 1.42E-04 | 0.024 | Altered group |
| CACNA1F | Xp11.23 | 1.04 | 1.22E-04 | 0.0226 | Unaltered group |
| PLAC1 | Xq26.3 | 1.18 | 1.69E-04 | 0.0256 | Unaltered group |
| SST | 3q27.3 | 1.3 | 9.03E-03 | 0.121 | Unaltered group |
